# A retrospective cohort study comparing differences in 30-day mortality among critically ill patients aged ≥ 70 years treated in European tax-based healthcare systems (THS) versus social health insurance systems

**DOI:** 10.1038/s41598-022-21580-y

**Published:** 2022-10-19

**Authors:** Bernhard Wernly, Hans Flaatten, Michael Beil, Jesper Fjølner, Raphael Romano Bruno, Antonio Artigas, Bernardo Bollen Pinto, Joerg C. Schefold, Malte Kelm, Sviri Sigal, Peter Vernon van Heerden, Wojciech Szczeklik, Muhammed Elhadi, Michael Joannidis, Richard Rezar, Sandra Oeyen, Georg Wolff, Brian Marsh, Finn H. Andersen, Rui Moreno, Sarah Wernly, Susannah Leaver, Ariane Boumendil, Dylan W. De Lange, Bertrand Guidet, Stefan Perings, Christian Jung

**Affiliations:** 1 Center for Public Health and Healthcare Research, Paracelsus Medical University of Salzburg, 5020 Salzburg, Austria; 2 Department of Internal Medicine, General Hospital Oberndorf, Teaching Hospital of the Paracelsus Medical University Salzburg, 5020 Salzburg, Austria; 3 Department of Clinical Medicine, University of Bergen, Bergen, Norway; 4 Department of Anaestesia and Intensive Care, Haukeland University Hospital, 5021 Bergen, Norway; 5 Deptartment of Medical Intensive Care, Faculty of Medicine, Hadassah Medical Center, Hebrew University of Jerusalem, 91120 Jersualem, Israel; 6 Department of Anesthesia and Intensive Care, Viborg Regional Hospital, 8800 Viborg, Denmark; 7 Department of Cardiology, Pulmonology and Vascular Medicine, Medical Faculty, Heinrich-Heine-University Duesseldorf, Moorenstraße 5, 40225 Düsseldorf, Germany; 8 Department of Intensive Care Medicine, CIBER Enfermedades Respiratorias, Corporacion Sanitaria Universitaria Parc Tauli, Autonomous University of Barcelona, 08208 Sabadell, Spain; 9 Department of Acute Medicine, Geneva University Hospitals, 1205 Geneva, Switzerland; 10 Department of Intensive Care Medicine, Inselspital, Universitätsspital, University of Bern, 3010 Bern, Switzerland; 11 Deptartment of Anesthesia, Intensive Care and Pain Medicine, Faculty of Medicine, Hadassah Medical Center, Hebrew University of Jerusalem, 91120 Jerusalem, Israel; 12 Center for Intensive Care and Perioperative Medicine, Jagiellonian University Medical College, 31-008 Krakow, Poland; 13 Faculty of Medicine, University of Tripoli, R6XF+46G, Tripoli, Libya; 14 Division of Intensive Care and Emergency Medicine, Department of Internal Medicine, Medical University Innsbruck, 6020 Innsbruck, Austria; 15 Clinic of Internal Medicine II, Department of Cardiology and Intensive Care, Paracelsus Medical University of Salzburg, 5020 Salzburg, Austria; 16 Department of Intensive Care 1K12IC, Ghent University Hospital, 9000 Ghent, Belgium; 17 Mater Misericordiae University Hospital, Dublin, D07 R2WY Ireland; 18 Department of Anaesthesia and Intensive Care, Ålesund Hospital, 6017 Ålesund, Norway; 19 Department of Circulation and Medical Imaging, Norwegian University of Science and Technology, 7491 Trondheim, Norway; 20 Centro Hospitalar de Lisboa Central, Lisbon, Portugal; 21 Faculdade de Ciências Médicas de Lisboa, Nova Medical School, Lisbon, Portugal; 22 Faculdade de Ciências da Saúde, Universidade da Beira Interior, Covilha, Portugal; 23 General Intensive Care, St. George´s University Hospital NHS Foundation Trust, London, SW17 0QT UK; 24 Inserm, Service de réanimation, Institut Pierre-Louis d’épidémiologie et de Santé Publique, Hôpital Saint-Antoine, AP-HP, Sorbonne Université, 184, Rue du Faubourg-Saint-Antoine, 75012 Paris, France; 25 Department of Intensive Care Medicine, University Medical Center, University Utrecht, 3584 CX Utrecht, The Netherlands

**Keywords:** Outcomes research, Epidemiology

## Abstract

In Europe, tax-based healthcare systems (THS) and social health insurance systems (SHI) coexist. We examined differences in 30-day mortality among critically ill patients aged ≥ 70 years treated in intensive care units in a THS or SHI. Retrospective cohort study. 2406 (THS n = 886; SHI n = 1520) critically ill ≥ 70 years patients in 129 ICUs. Generalized estimation equations with robust standard errors were chosen to create population average adjusted odds ratios (aOR). Data were adjusted for patient-specific variables, organ support and health economic data. The primary outcome was 30-day-mortality. Numerical differences between SHI and THS in SOFA scores (6 ± 3 vs. 5 ± 3; *p* = 0.002) were observed, but clinical frailty scores were similar (> 4; 17% vs. 14%; *p* = 0.09). Higher rates of renal replacement therapy (18% vs. 11%; *p* < 0.001) were found in SHI (aOR 0.61 95%CI 0.40–0.92; *p* = 0.02). No differences regarding intubation rates (68% vs. 70%; *p* = 0.33), vasopressor use (67% vs. 67%; *p* = 0.90) and 30-day-mortality rates (47% vs. 50%; *p* = 0.16) were found. Mortality remained similar between both systems after multivariable adjustment and sensitivity analyses. The retrospective character of this study. Baseline risk and mortality rates were similar between SHI and THS. The type of health care system does not appear to have played a role in the intensive care treatment of critically ill patients ≥ 70 years with COVID-19 in Europe.

## Introduction

This study aimed to investigate clinical differences between the two predominant European health care systems in critically ill COVID-19 patients aged 70 years and older. The classification into national health care systems, social insurance systems, and private insurance systems is often used to compare the performance of different national organizational forms of health care systems^1–5^. In Europe a rough dichotomization can be made between two general health care systems, the tax-based healthcare system (THS) and the social health insurance system (SHI)^2,3,6^. The World Health Organization defines the THS as a system in which more than half of public health expenditures are financed by revenues other than earmarked payroll taxes (this in contrast to SHI) and in which access to publicly financed services is at least formally open to all citizens^1,5^. In an analysis of the two very old intensive care patients (VIP) studies, we recently highlighted differences in admission policies, as well as management and outcomes in critically ill elderly patients between the two predominant organizational forms of health care systems in Europe^7^. To the best of our knowledge, there are no studies that have examined the effects of THS versus SHI on elderly, critically ill patients with COVID-19, and this knowledge gap motivated us to conduct this analysis.

The coronavirus disease (COVID-19) pandemic has put healthcare systems worldwide to an involuntary stress test and pushed critical care capacities to their limits even in highly developed health systems. As a result, some countries have been forced to develop criteria for prioritizing patients who benefit most from intensive care and to restrict access, at least temporarily, to patients who are unlikely to benefit from these medical interventions in terms of mortality and morbidity^8^. With increasing age, the benefit of intensive care treatment has been the subject of scientific, clinical, and ethical debates even before COVID-19^9^. Especially in older patients, who are not only acutely critically ill but also more vulnerable overall, the added prognostic value of critical care *quoad vitam* is unclear^9–12^. The assessment of frailty has been established for a structured assessment of the vulnerability of critically ill patients and can be quantified by means of the Clinical Frailty Scale (CFS). We have already confirmed the prognostic relevance of the CFS in critically ill patients aged 80 years and older in terms of survival in several prospective studies^11–13^.

Our primary objective was to examine differences in 30-day mortality among critically ill patients aged ≥ 70 years treated in intensive care units in a THS or SHI.

## Methods

### Setting

A retrospective cohort study of the COVIP study (COVID-19 in very old intensive care patients) which is part of the Very old Intensive care Patients (VIP) project (www.vipstudy.org) was performed. Baseline characteristics, treatment, and outcomes of patients, in intensive care units from countries classified as THS and SHI, respectively, were compared. The statistical methodology was performed similarly to a preliminary work from the VIP study^14^. The COVIP study was endorsed by the European Society of Intensive Care Medicine (ESICM) and registered on ClinicalTrials.gov (NCT04321265). The official study protocol is available online (https://vipstudy.org/covip-study/). The COVIP study complies with the European Union General Data Privacy Regulation (GDPR) directive, which applies in most participating countries. As in the previous VIP-studies, national coordinators recruited intensive care units (ICU), coordinated national and local ethical permissions, and supervised patient recruitment at the national level. A full list of all Collaborators is provided in Supplementary Table 1.

### Participants

Critically ill patients aged 70 years and older admitted for COVID-19 to an ICU were included. The decision to admit to an ICU was made by the attending physicians. Patients were classified as having been treated in a THS (Denmark, Ireland, Italy, Norway, Spain, Portugal, United Kingdom) or SHI (Austria, Belgium, France, Germany, Netherlands, Poland, Romania).

### Eligibility criteria

All patients admitted between 19th March 2020 and 4th February 2021 to one of the participating 129 ICUs were included. Only patients with complete data on age, sex, clinical frailty score (CFS), sequential organ failure assessment (SOFA) score and 30-day-mortality were included (Supplementary Fig. 1), leaving no missing values on these variables. Other missing values were handled by listwise deletion. Data collection started on ICU admission. The admission day was defined as day one, all consecutive days were numbered sequentially from the admission date. The methodology was performed similarly to a preliminary work from the VIP study^14^.

### Outcomes, exposures, predictors and confounders

The primary endpoint of this study was 30-day-mortality, the secondary endpoints were ICU- and 90-day-mortality, and rates of treatment limitation (the frequency of decisions to withdraw or withhold treatment). The primary exposure was being in a SHI versus THS. We used the dichotomisation chosen by the World Bank and the Organisation for European Economic Co-operation (OEEC)^2^.

For each patient relevant baseline characteristics (including age, sex, main reason for admission, and frailty) and management strategies (including use of renal replacement therapy (RRT), mechanical ventilation (MV), non-invasive ventilation (NIV), and rates of vasoactive use) were documented. Also, any treatment limitations (treatment withheld, or treatment withdrawn) were documented. Frailty was assessed by the Clinical Frailty Score (CFS) and the respective visual and simple descriptions were used with permission^15,16^. The gross domestic product (GDP) per capita for 2019 in US$ was accessed from the International Money Fund (IMF), the human development index (HDI) from the United Nations Development Program (UNDP)^17^, and the total (compulsory, voluntary, out-of-pocket) amount of health spending per capita in US$ in 2019 from the Organisation for Economic Co-operation and Development (OECD)^18^. The methodology was performed similarly to a preliminary work from the VIP^14^.

### Statistical analysis

The study size was defined by the retrospective dataset and no sample size calculation was performed. The data are likely to be clustered on an ICU level. Therefore, a multilevel regression approach was chosen to correct for potential confounders and bias. As the health economic data do not vary within a given cluster, generalised estimation equations (GEE) with robust standard errors to produce population average odds ratios were used. The survival analysis/GEE-based analyses were conducted using only robust estimators of the standard errors and not in the sense of robustness against violations of normality assumptions as for the robust methods (e.g., Mann–Whitney tests) used for the univariate analyses. Model-1 includes only the ICU as a panel. Model-2 includes patient-specific confounders (sex, age per year, SOFA score per point, frailty score per CFS point). Model-3 adds the health economic confounders (HDI). Adjusted odds ratios (aOR) and respective 95% confidence intervals (95%CI) were obtained. Sensitivity analyses stratifying 30-day-mortality for patient-specific characteristics, treatment strategies and health economic data and local 7-day-incidences were performed. Continuous data are given as median ± inter-quartile range (IQR) and compared using Mann’s Whitney U-Test or mean ± standard deviation (SD) and compared using Student’s T-Test accordingly. Categorical data are given as numbers (percentage) and compared using the chi-square test. All tests were two-sided, and a *p*-Value of < 0.05 was considered statistically significant. Stata/IC 17 was used for all statistical analyses. The statistical methodology was performed similarly to a preliminary work from the VIP study^14^.

### Ethics approval

Each study site obtained approval from the institutional research ethics board (IRB). The original vote was obtained at the Ethics Committee of the Medical Faculty of Heinrich-Heine-University Düsseldorf (Vote Number #2020-892). In some participating countries, patients could be recruited without informed consent, whereas in other countries, informed consent had to be obtained because of the respective ethical approval procedures. In all countries where informed consent was deemed necessary by the respective ethics committee, informed consent was obtained from all participating individuals. We confirm that all methods were performed in accordance with the relevant guidelines and regulations.

## Results

### Baseline demographics in SHI versus THS

In total, we included 2406 patients in this analysis. Patients were defined as being in a THS (n = 886) or SHI (n = 1520) health care system. The mean age was relatively similar in both groups (76 vs. 75 years; *p* = 0.08), while in the THS group fewer patients were between 70 and 79 years old (80 vs. 84%) and more patients were between 80 and 89 years old (19 vs. 15%; *p* = 0.03). We found no differences regarding patient´s sex and BMI (see Table 1). In the THS group SOFA scores at baseline were significantly higher (6 vs. 5 pts.; *p* = 0.002), whereas no significant difference was observed regarding CFS and length of stay (see Table 1). More patients in the THS group suffered from ischemic heart disease (24 vs. 18%; *p* = 0.005), whereas no difference was shown for all other comorbidities (see Table 1). Table 2 provides an overview of the number of patients from each country enrolled in this study. A higher health spending per capita (median 5.376 vs. 3.616 US$; *p* < 0.001), HDI (median 0.904 vs. 0.901; *p* < 0.001) and GDP (median 41.897 vs. 29.993 US$; *p* < 0.001) were observed in the THS group. We found no differences in days in the hospital prior to ICU admission and days with symptom onset prior to hospital admission between THS and SHI (Table 1).

**Table 1:** Demographic and clinical characteristics of the cohort.

	THS	SHI	*p*-Value
	(n = 1520)	(n = 886)	
**Sex**			
Male—n (%)	1094 (72%)	615 (69%)	0.19
Female—n (%)	426 (28%)	271 (31%)	
Age—years			
Mean (SD)	76 (5)	75 (4)	0.08
**Age categories**			
Age 70–79 years	1,217 (80%)	747 (84%)	0.03
Age 80–89 years	291 (19%)	136 (15%)	
Age ≥ 90 years	10 (1%)	3 (< 1%)	
**BMI—kg/m**^**2**^			
Mean (SD)	28 (5)	28 (5)	0.16
**SOFA score—points**			
Mean (SD)	6 (3)	5 (3)	0.002
SOFA > 5 pts.—n (%)	670 (44%)	349 (39%)	0.02
**Clinical frailty scale—points**			
Mean (SD)	3 (2)	3 (1)	0.35
CFS > 4 pts. n (%)	254 (17%)	124 (14%)	0.09
**Length of stay—days**			
Median (IQR)	11 (6–22)	13 (6–23)	0.11
**Concomitant diseases**			
Diabetes mellitus—n (%)	514 (34%)	278 (31%)	0.24
Ischemic heart disease—n (%)	362 (24%)	163 (18%)	0.005
Chronic kidney disease—n (%)	246 (16%)	139 (16%)	0.48
Arterial hypertension—n (%)	998 (66%)	579 (65%)	0.74
Chronic pulmonary disease—n (%)	347 (23%)	191 (22%)	0.69
Chronic heart failure—n (%)	230 (15%)	109 ()	0.16

*IQR* interquartile range, *SD* standard deviation, *SHI* social health insurance system, *SOFA* sequential organ failure assessment, *THS* tax-based health system.

**Table 2:** Healthcare economical characteristics of the cohort.

	THS	SHI	*p*-value
	(n = 1520)	(n = 886)	
**Country**			
Austria—n (%)	37 (2)		
Belgium—n (%)	174 (11)		
France—n (%)	680 (45)		
Germany—n (%)	231 (15)		
Netherlands—n (%)	283 (19)		
Poland—n (%)	113 (7)		
Rumania—n (%)	2 (0)		
Denmark—n (%)		212 (24)	
Ireland—n (%)		18 (2)	
Italy—n (%)		6 (1)	
Portugal—n (%)		88 (10)	
Spain—n (%)		360 (41)	
United Kingdom—n (%)		202 (23)	
**Health spending per capita (US$)**			
Median (IQR)	5376 (5376–5765)	3616 (3616–5276)	< 0.001
Proportion > median—n (%)	725 (48)	212 (24)	< 0.001
**HDI**			
Median (IQR)	0.904 (0.901–0.944)	0.901 (0.900–0.940)	< 0.001
Proportion > median—n (%)	725 (48)	432 (49)	0.64
**GDP per capita (US$)**			
Median (IQR)	41,897 (41,897–46,473)	29,993 (29,993–59,770)	< 0.001
Proportion > median n (%)	725 (48)	432 (49)	0.64

*GDP* gross domestic product, *HDI* human development index, *IQR* interquartile range, *SHI* social health insurance system, *THS* tax-based health system.

### Organ support and management

In the univariate analysis, the rates of vasopressor use (67% in both groups; *p* = 0.9) and invasive mechanical ventilation (68 vs. 70%; *p* = 0.33) were similar between both groups (see Table 3). These findings persisted after multivariable adjustment (see Table 4). Non-invasive ventilation was performed more frequently in the SHI group (32 vs. 21%; *p* < 0.001). However this association was not confirmed in the multivariable analysis (aOR 1.47 95%CI 0.89–2.42; *p* = 0.13). Yet, the rates of RRT were significantly higher in the SHI group (17 vs. 10%; *p* < 0.001) and remained so after multivariable adjustment (aOR 0.61, 95%CI 0.40–0.92; *p* = 0.02; Table 4).

**Table 3:** Univariate comparison of organ support, treatment limitations and mortality in THS versus SHI.

	THS	SHI	*p*-value
	n = 1520	n = 886	
**Organ support n (%)**			
Mechanical ventilation	1040 (68)	623 (70)	0.33
Vasoactive medication	1016 (67)	594 (67)	0.9
Renale replacement therapy	255 (17)	91 (10)	< 0.001
Non-invasive ventilation	317 (21)	283 (32)	< 0.001
**Treatment limitation n (%)**			
Any treatment limitation	600 (40)	337 (39)	0.37
Treatment withholding	510 (34)	270 (31)	0.09
Treatment withdrawal	280 (19)	204 (23)	0.01
**Mortality n (%)**			
ICU mortality	666 (44)	404 (46)	0.41
30-day-mortality	713 (47)	441 (50)	0.16
90-day-mortality	779 (55)	477 (57)	0.22

Categorical variables were compared using Chi-square.

*ICU* intensive care unit, *SHI* social health insurance system, *THS* tax-based health system.

**Table 4:** Generalised estimation equations (GEE) producing population average odds ratios.

	Model-1	Model-2	Model-3
	aOR (95%CI; *p*-value)	aOR (95%CI; *p*-value)	aOR (95%CI; *p*-value)
**Organ support**			
Mechanical ventilation	1.00 (0.65–1.53; *p* = 0.98)	1.01 (0.66–1.55; *p* = 0.96)	1.01 (0.66–1.56; *p* = 0.95)
Vasoactive medication	0.92 (0.64–1.32; *p* = 0.63)	0.91 (0.63–1.32; *p* = 0.62)	0.93 (0.64–1.34; *p* = 0.69)
Renale replacement therapy	0.47 (0.32–0.71; *p* < 0.001)	0.59 (0.39–0.91; *p* = 0.02)	0.61 (0.40–0.92; *p* = 0.02)
Non-invasive ventilation	1.41 (0.85–2.34; *p* = 0.18)	1.46 (0.88–2.42; *p* = 0.14)	1.47 (0.89–2.42; *p* = 0.13)
**Treatment limitation**			
Any treatment limitation	0.93 (0.67–1.29; *p* = 0.66)	1.02 (0.69–1.51; *p* = 0.93)	1.02 (0.69–1.50; *p* = 0.93)
Treatment withholding	0.80 (0.56–1.15; *p* = 0.23)	0.91 (0.59–1.41; *p* = 0.68)	0.91 (0.59–1.41; *p* = 0.68)
Treatment withdrawal	1.28 (0.89–1.84;* p* = 0.18)	1.40 (0.92–2.13; *p* = 0.12)	1.42 (0.95–2.12; *p* = 0.09)
**Mortality**			
ICU mortality	0.99 (0.76–1.30; *p* = 0.95)	1.10 (0.81–1.48; *p* = 0.54)	1.08 (0.80–1.46; *p* = 0.62)
30-day-mortality	1.07 (0.82–1.40; *p* = 0.63)	1.20 (0.88–1.63; *p* = 0.25)	1.18 (0.87–1.61; *p* = 0.29)
90-day-mortality	1.09 (0.80–1.47; *p* = 0.59)	1.21 (0.85–1.73; *p* = 0.28)	1.20 (0.84–1.72; *p* = 0.31)

Model-1: GEE with ICU as random effect and THS versus SHI as fixed effect.

Model-2: Model-1 plus patient level data (age, sex, SOFA-score and CFS).

Model-3 Model-2 plus health economic data (HDI).

Model-1 includes only the ICU as panel. Model-2 includes patient specific factors (sex, age per year, SOFA score per point, frailty score per CFS point and the admission diagnosis). Model-3 adds the HDI. Adjusted odds ratios (aOR) and respective 95% confidence intervals (95%CI) were obtained.

*CFS* clinical frailty scale, *HDI* human development index, *ICU* intensive care unit, *SOFA* sequential organ failure assessment.

### Mortality and treatment limitation analysis

**Figure 1:**
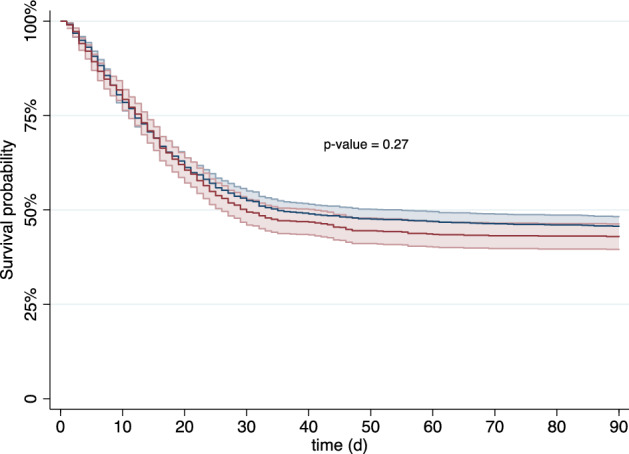
Kaplan–Meier curve depicting survival in THS (red) and SHI (blue) patients (with 95% CI). Abbreviations: CI: confidence interval; d: days; SHI: social health insurance system; THS: tax-based health system.

ICU mortality, as well as mortality at 30 and 90 days was similar in both groups (ICU: 44 vs. 46%, *p* = 0.41; 30-day: 47 vs. 50%, *p* = 0.16; 90-day: 55 vs. 57%, *p* = 0.22). This finding persisted after multivariable adjustment across all models (ICU: aOR 1.08 95%CI 0.80–1.46, *p* = 0.62; 30-day: aOR 1.18 95%CI 0.87–1.61, *p* = 0.29; 90-day: aOR 1.20 95%CI 0.84–1.72, *p* = 0.31). These findings are shown in Tables 3 and 4, and in the Kaplan Meier Curve in Fig. 1. The rates of any treatment limitations were similar between THS and SHI (40% vs. 39%; *p* = 0.37) and remained so after multivariable adjustment (aOR 1.02; 95%CI 0.69–1.50; *p* = 0.93). No significant difference was observed for withholding treatment (THS 34 vs. SHI 31%; *p* = 0.09; aOR 0.91 95%CI 0.59–1.41, *p* = 0.68). There was a trend towards lower rates of treatment withdrawal in THS (19% vs. 23%; *p* = 0.01) in the univariate analysis, however we could not observe this difference after multivariable adjustment (aOR 1.42; 95%CI 0.95–2.12; *p* = 0.09).

### Sensitivity analyses

**Figure 2:**
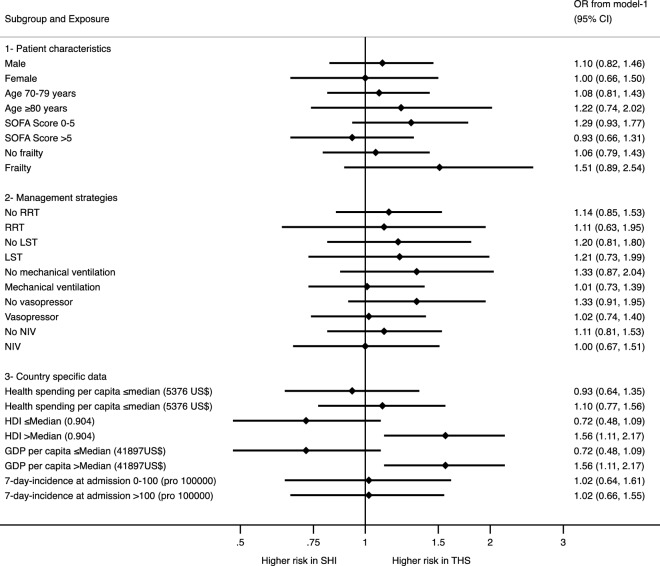
Sensitivity analyses stratifying 30-day-mortality in subgroups for patient-specific characteristics using generalised estimation equations (GEE) producing population average odds ratios. The depicted aORs from model-1 include only the ICU as panel. Abbreviations: aOR: adjusted odds ration; GDP: gross domestic product; HDI: human development index; ICU: intensive care unit; LST: life-sustaining treatment; NIV: non-invasive ventilation; SHI: social health insurance system; SOFA: sequential organ failure assessment; THS: tax-based health system.

In the sensitivity analyses comparing 30-day-mortality between SHI and THS for patient-specific characteristics, treatment strategies and health economic data (see Forest Plot in Fig. 2), we found that only in the subgroup of patients treated in countries with a HDI above the median of 0.904 (aOR 1.56 95%CI 1.11–2.17) and GDP per capita above the median of 41,892 US$ (aOR 1.56 95%CI 1.11–2.17), treatment in a THS was associated with higher odds for non-survival at 30 days.

## Discussion

This study explored differences in critically ill older patients suffering from COVID-19 between SHI and THS. We found no clinically relevant differences with regards to demographics and geriatric characteristics, such as frailty and comorbidities, as well as management strategies and outcomes.

A key point for the interpretation of this study is the categorization of health care systems. As discussed in our preliminary study, we are aware that the division of national health care systems into two distinct systems may be an over-simplification. In addition to this rough dichotomization of health care systems in Europe, there are also more sophisticated classification systems with higher granularity that can be used to subdivide the organization of health systems^3,19,20^. Some authors use a deductive approach and describe up to 27 different theoretically possible subtypes of health systems^21–23^. Ultimately, we consider all subclassifications to be variations of the original used subdivision here. These are, on the one hand, primarily tax-financed, municipally organized systems with a "single payer" (THS), or in a social insurance system (SHI) financed by predefined and earmarked premiums, mostly from payroll taxes, and organized rather pluralistically, where private and public service providers often coexist and compete. In addition to previously published scientific analyses, the World Bank and the Organization for European Economic Cooperation (OEEC) also use the pragmatic view of existing European health systems used in this study^2,18^. Apart from national health care systems, other organizing factors, such as equipment, management of ICUs may have influenced our results, although we do not have specific information on this. In the following, we will put the results of our study in context, and we believe that one of the strengths of our study is its relative uniqueness.

### Baseline risk distribution in SHI versus THS

In our study, some statistically significant differences in baseline characteristics were found between the SHI and THS groups, but we consider these to be clinically irrelevant given the small absolute numeric differences. Triage for ICU admission is a difficult issue with wide variation in perception and acceptance, not only across countries^10,24–27^. However, when baseline characteristics are used as a possible surrogate parameter on different admission policies between the two systems, there is no evidence of such a difference based on our data.

These findings contrast with the results of our recent analysis in which we compared critically ill elderly (80 years and older) patients in SHI and THS with respect to admission characteristics, organ replacement procedures, and outcomes before the COVID-19 pandemic. In this study, we detected indirect evidence of a more liberal admission policy in SHI, indicated by higher age and frailty scores^7^. However, we believe that even in the absence of a pandemic, the differences between the two health systems across different European countries might be subtle in nature. Yet, because the COVID-19 pandemic has strained health systems of all types, we believe this has offset and compensated for systemic differences. Since we also included younger patients (70 years and older) compared to our last study, an exact comparison of the age structures between the two studies is impossible^7^. However, it is striking that the number of patients over 90 years of age in the COVIP study was very low. We interpret this as a possible indication of stricter admission criteria across all ICUs, countries and health systems.

### Organ support and management in SHI versus THS

In this analysis, no differences between SHI and THS regarding invasive ventilation and the use of vasopressors was shown. However, it is interesting to note that, as in our earlier study, RRT was used significantly more frequent in SHI. This is of interest as this extra effort did not result in any survival benefit. Since the rate of patients with preexisting renal disease was similar between SHI and THS, and median SOFA scores were only slightly different, one could speculate here whether the more aggressive use of RRT in SHI was triggered by financial motivation, but also clinical training in health care settings or used more liberally due to less financial restrictions. Of note, the renal SOFA component was similar in SHI and THS (*p* = 0.52). These findings fit well with the STARRT-AKI study and a recent meta-analysis that showed no benefit for earlier use of RRT^28,29^.

### Mortality analysis

In brief, we did not detect a mortality in critically ill patients over 70 years between SHI and THS. Mortality was similar at the time of ICU discharge, 30 days, and 90 days, even after multivariable adjustment for clinical and health economic data, as well as clinical management. Likewise, extensive sensitivity analyses did not reveal a clinically relevant difference between the two health care systems. Of note, also in patients above 80 years of age, reflecting the age group of the VIP studies, we found no differences. We also found no evidence of disparate mortality in the sensitivity analyses related to incidence in the population at the time of admission. There was a signal towards higher mortality in THS with higher GDP and HDI (GDP and HDI overlapped in this analysis). However, we interpret this lone signal for differential mortality primarily in the context of multiple testing and do not wish to generate a hypothesis based on it.

### Limitations

This is a retrospective cohort study and is therefore subject to the limitations associated with this methodology. No sample size calculation was performed for this analysis, therefore, the absence of differences between groups could be due to an underpowered study to detect very subtle differences in outcomes between the study groups. Like our previous study, this study does not directly provide information on the triage process and prescreening prior to actual ICU admission. Also, this study is applicable only to patients aged between 70 and 90, as only very few patients above 90 years were included. Another weakness of this analysis is also that different important countries using a THS were not included in our database. In particular, Sweden, which has chosen a special path in the pandemic anyway, is missing. Local COVID-19 incidences play another factor of uncertainty. Although the average number of new infections and thus the burden of critically ill patients may be manageable in a given country, whereas the occurrence of local clusters and excessive strain on health care systems is typical for the course of COVID-19. Our analysis took into account the 7-day incidence at the country level in a sensitivity analysis—nevertheless, clusters at the local level may have influenced the results. However, given that the overall results of this study were very homogeneous, we do not believe that we have overlooked relevant epidemiological factors of the pandemic in this regard. Finally, it must be emphasized that other factors, including nationally distributed ones, may have influenced our results, such as the local organization of ICUs, cultural factors, or the availability of innovative therapies.

## Conclusion

The aims of this study were to compare the admission characteristics, treatment strategies (frequency of organ replacement procedures and therapy target limitations) and mortality at three time points (time of ICU discharge, after 30 and after 90 days) of critically ill patients aged between 70 and 90 admitted to an ICU due to severe COVID-19 disease between Bismarck systems and Beveridge systems in Europe. We found no evidence of clinically relevant differences in admission characteristics between SHI systems and THS. With regard to intensive care management, there was evidence of more frequent use of renal replacement therapies in SHI systems compared to THS. There were no indications of differences between SHI systems and THS with regard to mortality—which may be considered the ultimate measure of performance, especially in intensive care medicine. It remains unknown to what extent the observed parity of intensive care for elderly patients with COVID-19 is a specific feature of critical care medicine or a sign of socioeconomic convergence of the two systems.

## Supplementary Information

Supplementary Information 1.

Supplementary Information 2.

## Data Availability

Individual participant data that underlie the results reported in this article (after the deidentification), the study protocol will be available immediately after publication to researchers who provide a methodologically sound proposal for individual participant data meta-analysis after approval of their proposal by the country coordinators and the COVIP steering group (available at https://vipstudy.org/covip-study/). The proposal should be directed to the Corresponding author and to gain access, data requestors will need to sign a data access agreement. The data will be transmitted via email.

## References

[1] Savedoff, W. Tax-based financing for health systems: Options and experiences. *Health System Financing, Expenditure and Resource Allocation* (2004).

[2] Wagstaff, A. Social health insurance vs. tax-financed health systems—evidence from the OECD. *Policy Res Work Pap*. 10.1596/1813-9450-4821 (2009).

[3] van der Zee J, Kroneman MW (2007). Bismarck or Beveridge: A beauty contest between dinosaurs. BMC Health Serv. Res..

[4] Or Z (2010). Are health problems systemic? Politics of access and choice under Beveridge and Bismarck systems. Health Econ. Policy Law.

[5] Doetinchem, O., Evans, G. C. & Evans, D. Thinking of introducing social health insurance? Ten questions. *World Health Report* (2010).

[6] Wendt C (2009). Mapping European healthcare systems: A comparative analysis of financing, service provision and access to healthcare. J. Eur. Soc. Policy.

[7] Wernly B (2021). Provision of critical care for the elderly in Europe: A retrospective comparison of national healthcare frameworks in intensive care units. BMJ Open.

[8] Guidet B, de Lange DW, Flaatten H (2017). Should this elderly patient be admitted to the ICU?. Intensive Care Med..

[9] Beil M (2020). On predictions in critical care: The individual prognostication fallacy in elderly patients. J. Crit. Care.

[10] Levy MM (2012). Triage decision making for the elderly. Crit. Care Med..

[11] Jung C (2021). The impact of frailty on survival in elderly intensive care patients with COVID-19: The COVIP study. Crit. Care Lond. Engl..

[12] Flaatten H (2017). The impact of frailty on ICU and 30-day mortality and the level of care in very elderly patients (≥ 80 years). Intensive Care Med..

[13] Flaatten H, deLange D, Jung C, Beil M, Guidet B (2021). The impact of end-of-life care on ICU outcome. Intensive Care Med..

[14] Wernly B (2021). Provision of critical care for the elderly in Europe: A retrospective comparison of national healthcare frameworks in intensive care units. BMJ Open.

[15] Rockwood K (2005). A global clinical measure of fitness and frailty in elderly people. CMAJ Can. Med. Assoc. J. J. Assoc. Med. Can..

[16] Katz S (1983). Assessing self-maintenance: Activities of daily living, mobility, and instrumental activities of daily living. J. Am. Geriatr. Soc..

[17] United Nations Development Program. Human Development Report 2020. http://hdr.undp.org/sites/default/files/hdr2020.pdf (2020).

[18] OECD. Health at a Glance. https://data.oecd.org/healthres/health-spending.htm (2020).

[19] Gaeta M (2017). An overview of different health indicators used in the European health systems. J. Prev. Med. Hyg..

[20] Herzlinger RE, Parsa-Parsi R (2004). Consumer-driven health care: Lessons from Switzerland. JAMA.

[21] Böhm K, Schmid A, Götze R, Landwehr C, Rothgang H (2013). Five types of OECD healthcare systems: Empirical results of a deductive classification. Health Policy.

[22] Rothgang H, Cacace M, Grimmeisen S, Wendt C (2005). 9 The changing role of the state in healthcare systems. Eur. Rev.

[23] Schieber, G. J. *Financing and delivering health care*. (Organisation for Economic Co-operation and Development, 1987).

[24] Guidet B (2018). Attitudes of physicians towards the care of critically ill elderly patients: A European survey. Acta Anaesthesiol. Scand..

[25] Guidet B (2017). Effect of systematic intensive care unit triage on long-term mortality among critically ill elderly patients in France: A randomized clinical trial. JAMA.

[26] Netters S (2021). Pandemic ICU triage challenge and medical ethics. BMJ Support. Palliat. Care.

[27] Wilkinson D, Zohny H, Kappes A, Sinnott-Armstrong W, Savulescu J (2020). Which factors should be included in triage? An online survey of the attitudes of the UK general public to pandemic triage dilemmas. BMJ Open.

[28] Bhatt GC, Das RR, Satapathy A (2021). Early versus late initiation of renal replacement therapy: Have we reached the consensus? An updated meta-analysis. Nephron.

[29] STARRT-AKI Investigators *et al.* Timing of initiation of renal-replacement therapy in acute kidney injury. *N. Engl. J. Med.* **383**, 240–251 (2020). 10.1056/NEJMoa2000741 32668114

