# Supplementary material for: A retrospective cohort study comparing differences in 30-day mortality among critically ill patients aged ≥ 70 years treated in European tax-based healthcare systems (THS) versus social health insurance systems
Source: Sci Rep. 2022 Oct 19;12:17460. doi: 10.1038/s41598-022-21580-y (PMC9580441; doi:10.1038/s41598-022-21580-y)
Supplement: Supplementary file 1 — Supplementary Information 1. [file 41598_2022_21580_MOESM1_ESM.pptx]

## Slide 1
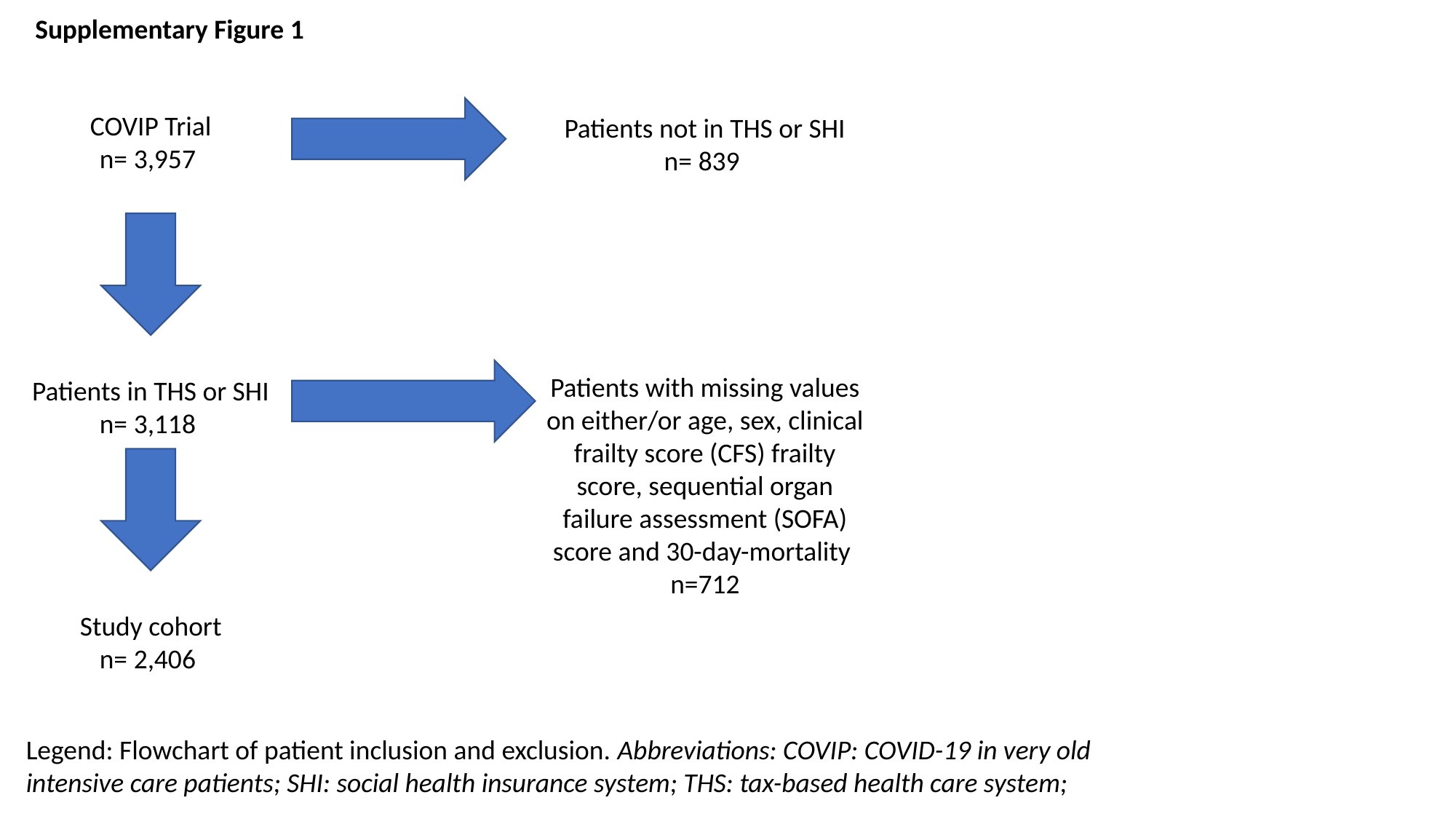

Supplementary Figure 1
COVIP Trial
n= 3,957
Patients not in THS or SHI
n= 839
Patients with missing values on either/or age, sex, clinical frailty score (CFS) frailty score, sequential organ failure assessment (SOFA) score and 30-day-mortality
n=712
Patients in THS or SHI
n= 3,118
Study cohort
n= 2,406
Legend: Flowchart of patient inclusion and exclusion. Abbreviations: COVIP: COVID-19 in very old intensive care patients; SHI: social health insurance system; THS: tax-based health care system;
